# Supplementary material for: Bio-Derived Carbon with Tailored Hierarchical Pore Structures and Ultra-High Specific Surface Area for Superior and Advanced Supercapacitors
Source: Nanomaterials (Basel). 2021 Dec 23;12(1):27. doi: 10.3390/nano12010027 (PMC8746562; doi:10.3390/nano12010027)
Supplement: Supplementary file 1 [file nanomaterials-12-00027-s001.zip › nanomaterials-1488627-supplementary.pdf]

Supporting Information

Bio-derived Carbon with Tailored Hierarchical Pore Structures and Ultra-high Specific Surface Area for Superior and Advanced Supercapacitors

Fuming Zhang[1], Xiangshang Xiao[1], Dayakar Gandla[1], Zhaoxi Liu[1], Daniel Tan[1,2*] and Yair Ein-Eli [2,3*]

1 Department of Materials Science and Engineering, Guangdong Technion - Israel Institute of Technology, 241 Daxue Road, Jinping District, Shantou, Guangdong 515063, China
2 Department of Materials Science and Engineering, Technion - Israel Institute of Technology, Haifa 3200003, Israel
3 Grand Technion Energy program (GTEP) Technion - Israel Institute of Technology, Haifa 3200003, Israel
* Correspondence: daniel.tan@gtiit.edu.cn(D.G.), eineli@technion.ac.il(Y.E-E.)

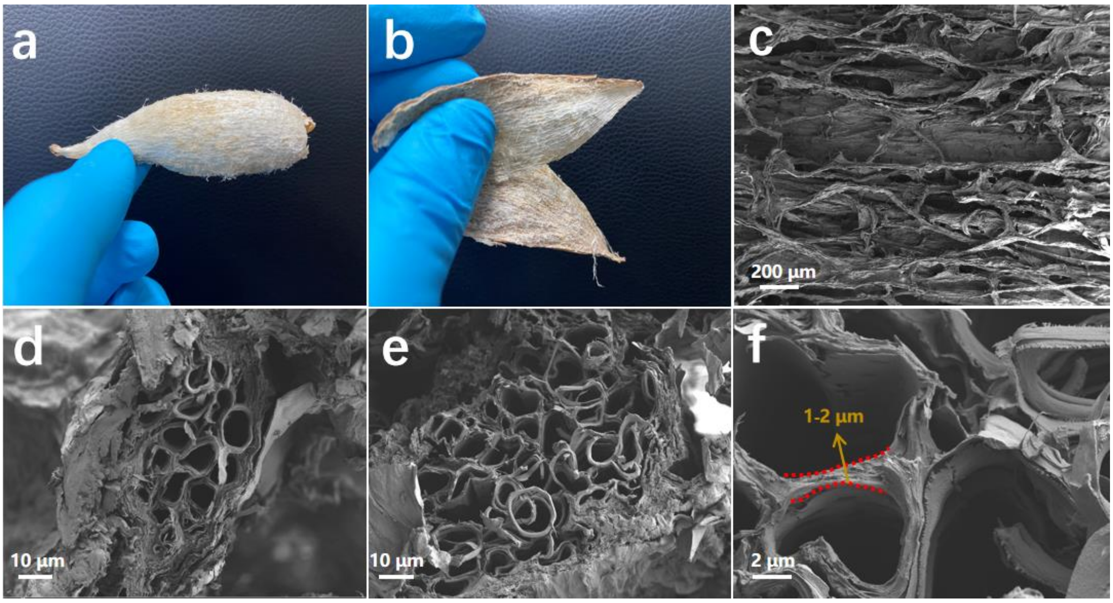

Figure S1. Digital photo a) and b) Metaplexis Japonica overall and surface morphology; SEM image of precursor c)-d) at different magnification.

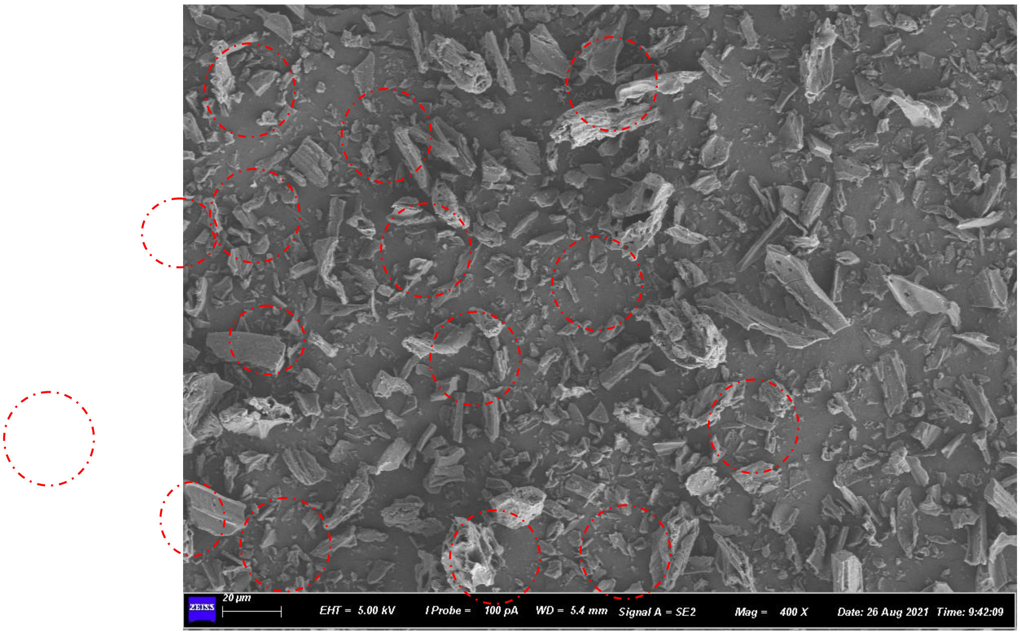

Figure S2. SEM image of MJ-5.

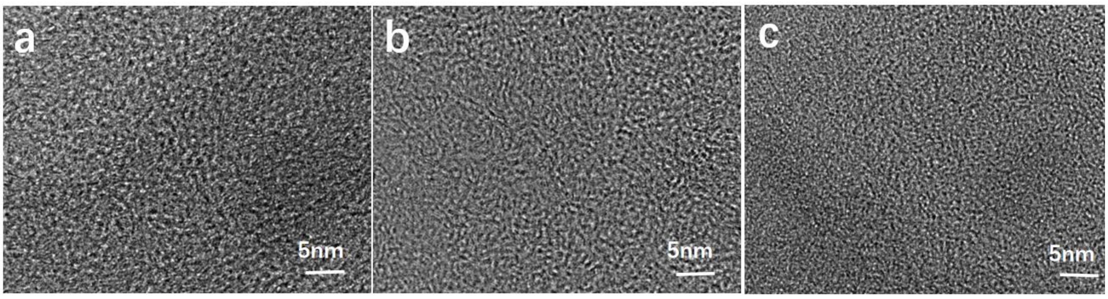

Figure S3. TEM image of a) MJ-1 b) MJ-3 c) MJ-5.

Table S1. Structural characteristics of the carbon materials.

Samples	S_{BET} ^{a}-total (m^2 g^{-1})	S_{micro}^{a} (m^2 g^{-1})	S_{meso}^{a} (m^2 g^{-1})	Pore volume-total (cm^3g^{-1})	Pore volume-meso(cm^3g-1)	C content (%)	O content (%)
YP-50	1868	1747	102	0.807	0.164	86.0	14.0
MJ-1	1917	1855	62	0.796	0.107	83.7	16.3
MJ-3	2783	2394	333	1.597	0.439	86.5	13.5
MJ-5	3635	2976	559	2.017	0.595	92.2	7.8

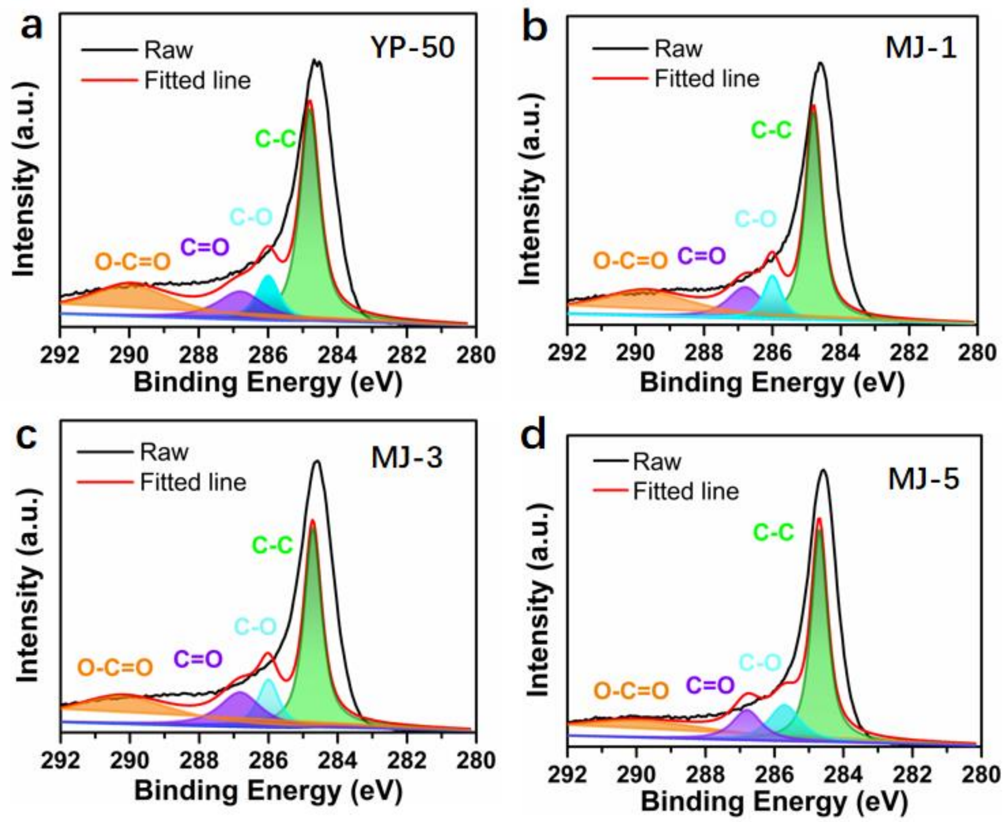

Figure S4. High-resolution C1s spectra of MJ-x and YP-50.

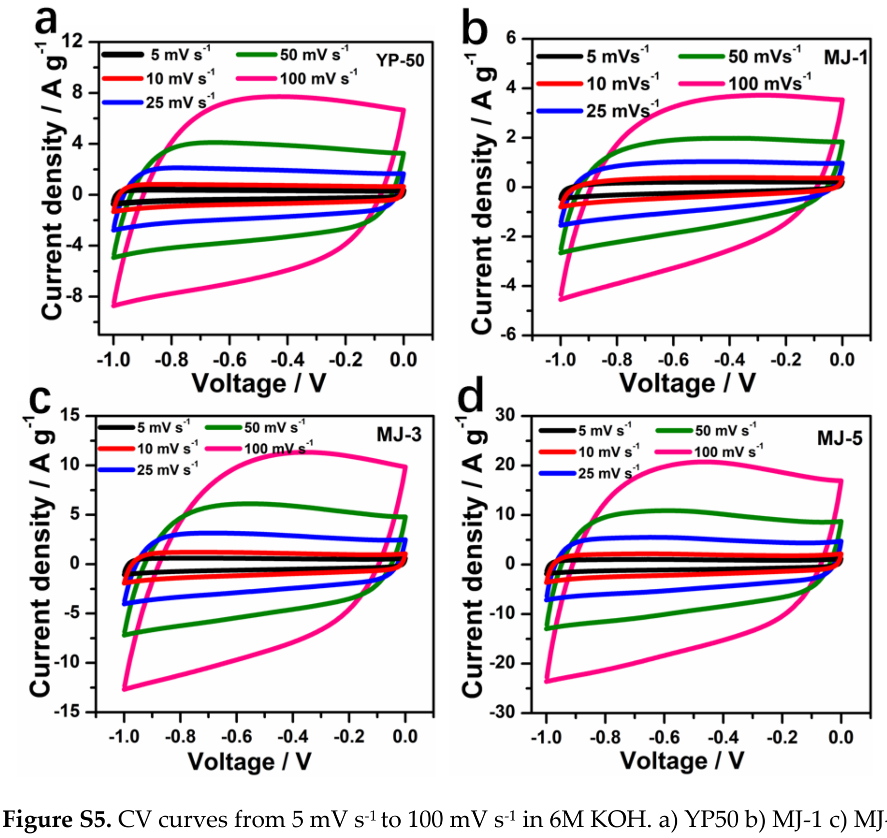

Figure S5. CV curves from 5 mV s^{-1} to 100 mV s^{-1} in 6M KOH. a) YP50 b) MJ-1 c) MJ-3 d) MJ-5.

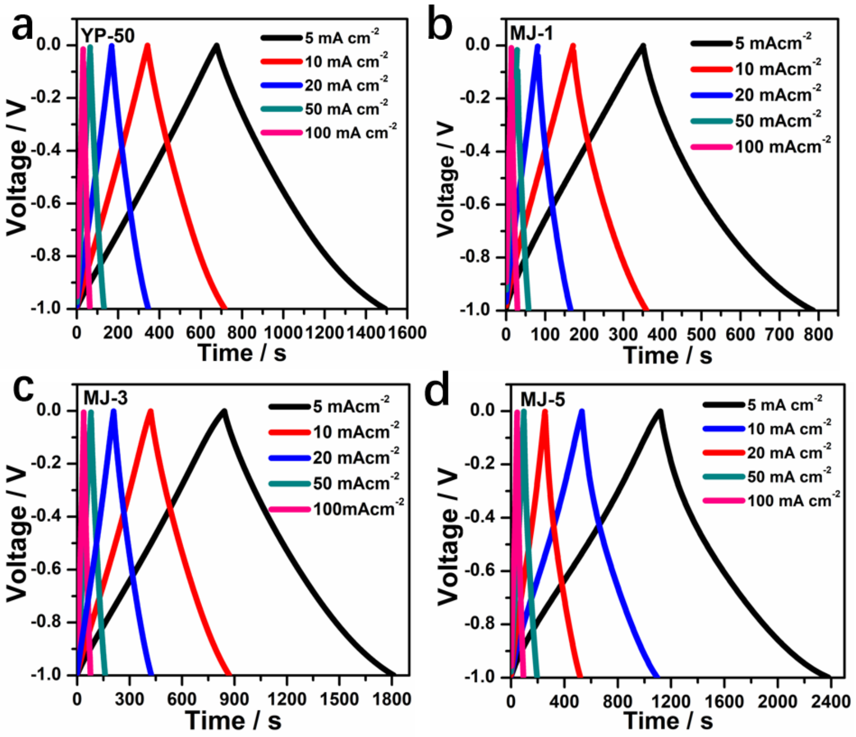

Figure S6. GCD plots from 5 mV s^{-1} to 100 mV s^{-1} in 6M KOH. a) YP50 b) MJ-1 c) MJ-3 d) MJ-5.

Table S2. The detailed ion size information.

DMP^+	SBP^+	TEA^+	BF4-	ACN
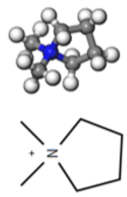	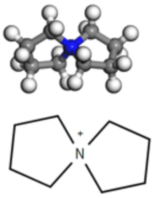	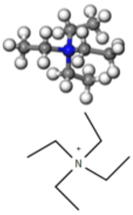	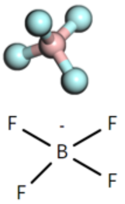	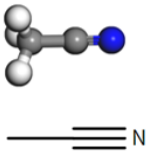
size/Å (from top to bottom is length, width and height)				
6.516	9.009	7.403	4.678	5.731
7.034	6.405	8.841	4.678	4.184
6.044	6.194	7.113	4.630	3.949
Van der Waals volume/$Å^3$				
117.29	143.30	162.08	49.27	45.55

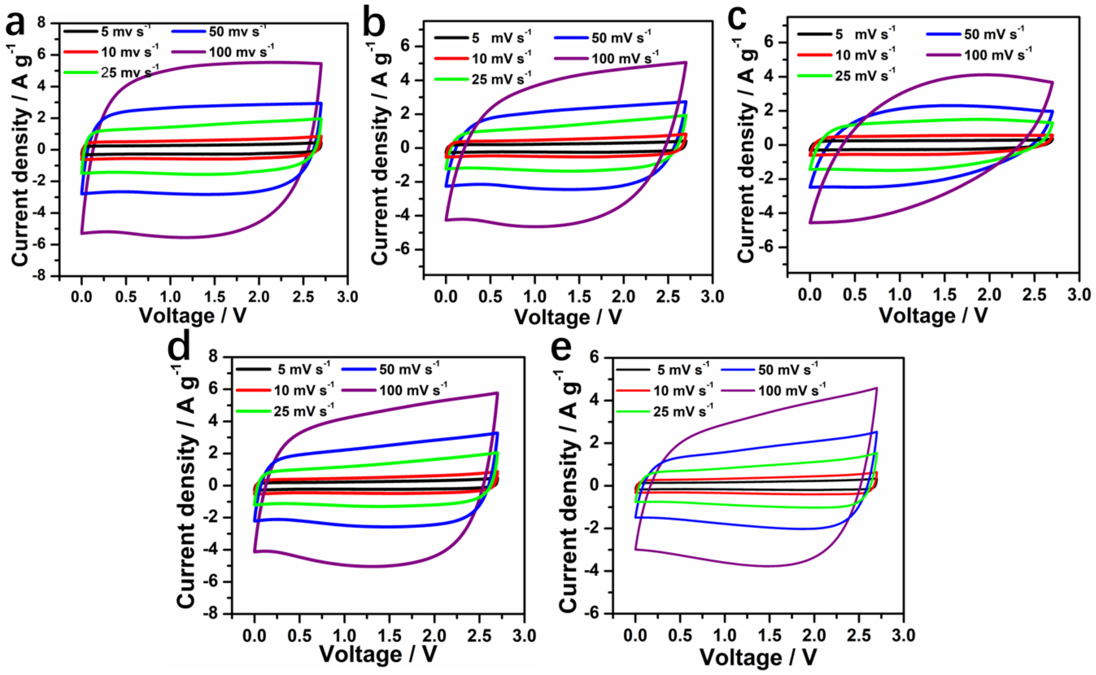

Figure S7. CV curves of MJ-x in different organic electrolyte**.** a) MJ-5 in $DMPBF_4$ b) MJ-5 in $SBPBF_4$ c) MJ-5 in $TEABF_4$ d) MJ-3 in $DMPBF_4$ e)MJ-1 in $DMPBF_4$.

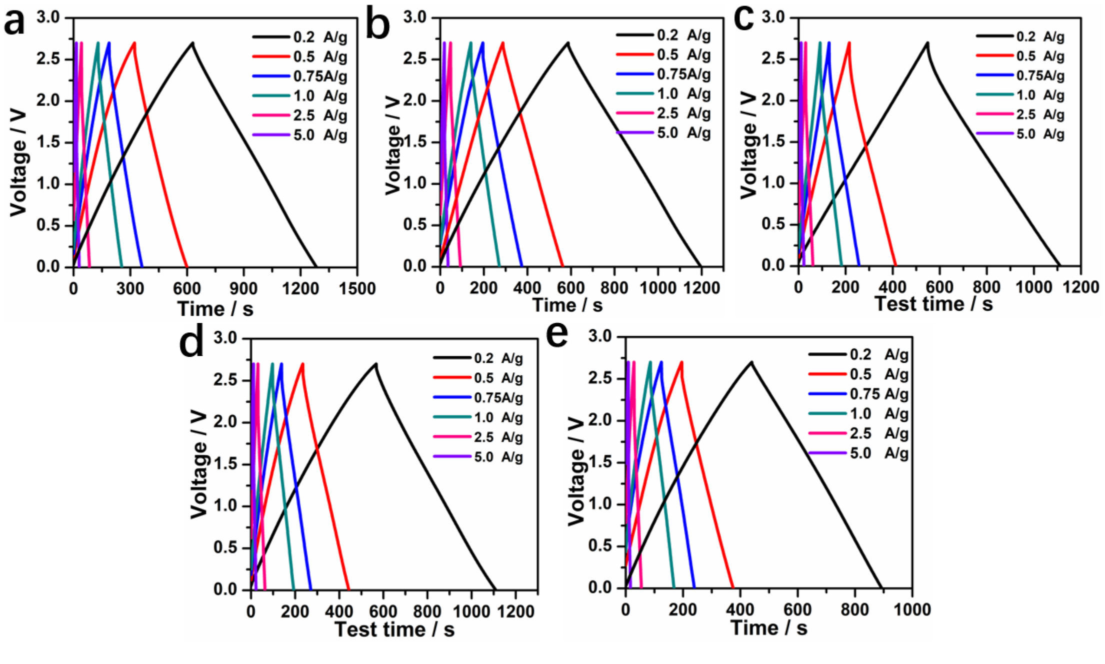

Figure S8. GCD plots of MJ-x in different organic electrolyte**.** a) MJ-5 in $DMPBF_4$ b) MJ-5 in $SBPBF_4$ c) MJ-5 in $TEABF_4$ d) MJ-3 in $DMPBF_4$ e) MJ-1 in $DMPBF_4$.

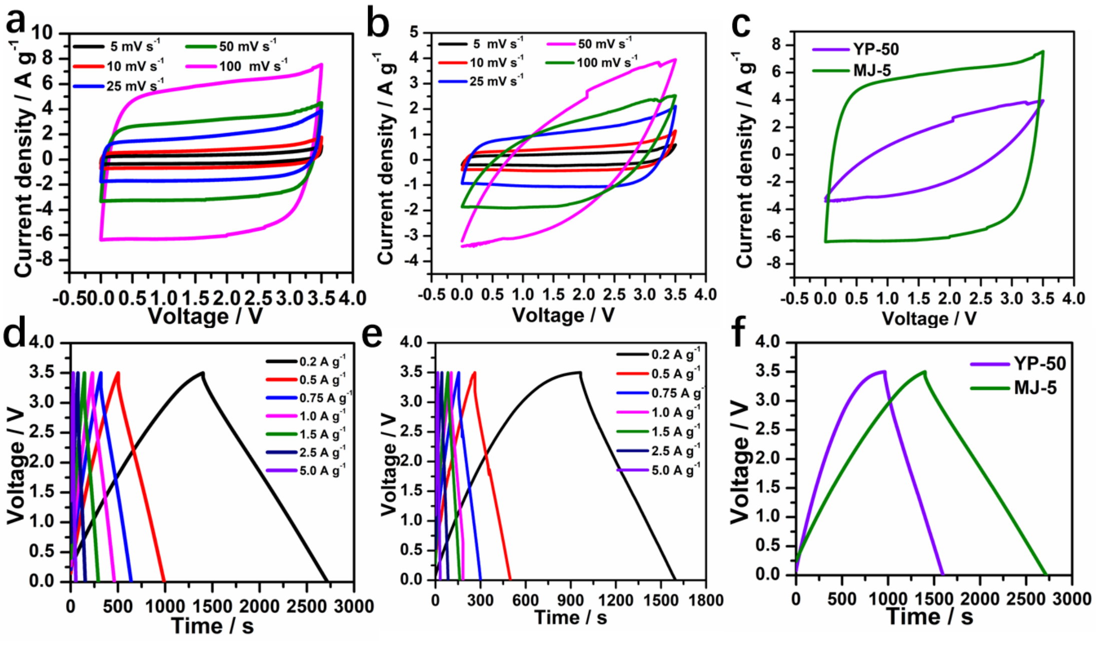

Figure S9. Electrochemical performance of MJ-5 and YP-50 in ionic liquid electrolyte**.** CV curves of a) MJ-5 b) YP-50 c) Comparison of CV curves at 100 mV s^{-1}; GCD plots of d) MJ-5 e) YP-50 f) Comparison of GCD plots at 0.2 A $g^{-1.}$
